# Heavy screen users are the heaviest among 10,000 children

**DOI:** 10.1038/s41598-019-46971-6

**Published:** 2019-08-01

**Authors:** Elina Engberg, Rejane A. O. Figueiredo, Trine B. Rounge, Elisabete Weiderpass, Heli Viljakainen

**Affiliations:** 1 Folkhälsan Institute of Genetics, Folkhälsan Research Center, Helsinki, Finland; 2 Department of Sports and Exercise Medicine, Clinicum, Faculty of Medicine, University of Helsinki, Helsinki, Finland; 3 Faculty of Medicine, University of Helsinki, Helsinki, Finland; 4 Department of Research, Cancer Registry of Norway, Institute of Population-based Cancer Research, Oslo, Norway; 5 International Agency for Research on Cancer, World Health Organization, Lyon, France; 6 Department of Food and Environmental Sciences, University of Helsinki, Helsinki, Finland

**Keywords:** Predictive markers, Risk factors

## Abstract

This cross-sectional study examined the associations of recreational screen time (viewing TV programs on any screen-based device and computer use, performed while sitting) with body mass index (BMI) categories and waist-to-height ratio (WHtR) tertiles in 10,228 children (mean age 11.1 years, SD 0.8). We categorized the children into Light, Medium and Heavy TV viewers and computer users, and into Low, Medium and High exercise groups. Compared with Light TV viewers, Medium (OR: 1.30, 95% CI: 1.11–1.52, when adjusted for age, sex, language, sleep duration and exercise) and Heavy (OR: 1.57, 95% CI: 1.34–1.83) TV viewers had a higher risk of being overweight. Similarly, Heavy computer users had a higher risk of being overweight (OR: 1.42, 95% CI: 1.21–1.67). We observed interactions between exercise and TV viewing (*p* = 0.012) or computer use (*p* = 0.010). However, Heavy TV viewers had a higher risk of being overweight in all exercise groups. The associations of TV viewing and computer use were similar with BMI and WHtR. To conclude, heavy sedentary screen time is associated with overweight and central adiposity in children. Moreover, heavy TV viewers have a higher risk for overweight and central adiposity, regardless of weekly exercise duration.

## Introduction

The alarming increase in childhood obesity is one of the most serious global health challenges of the 21^st^ century. The worldwide prevalence of overweight and obesity in children and adolescents has increased enormously during the last three or four decades, including a threefold increase in Finland^1,2^. Lifestyle factors, such as low levels of physical activity and a greater amount of time spent in sedentary behaviors have been suggested to contribute to the present obesity epidemic^3,4^. About one third of 9 to 15 year old Finnish children meet the physical activity guidelines for their age: moderate- or vigorous-intensity physical activity for one hour or more per day^5^. Furthermore, Finnish children spend more than half of their waking hours sitting or lying down, and 55% report more than two hours of screen time on at least five days per week^5^. Physical activity and sedentary behaviors are independent behaviors and may affect health through different mechanisms, but are also likely to be interrelated. It is easier to achieve higher levels of physical activity if time is taken from other common activities, such as screen time. Yet, the research evidence shows only small associations between moderate-to-vigorous physical activity and sedentary behaviors, therefore, high levels of both screen time and physical activity may co-exist^6^.

More time spent sitting is associated with poorer metabolic health and an increased risk for overweight and obesity in several adult populations^7^. Research evidence, mostly derived from observation studies, indicates that greater time spent in sedentary behaviors is also related to poorer cardiometabolic health and higher weight or adiposity in younger populations^8–10^. The existing research evidence shows associations between higher levels of TV viewing or total screen time and unfavorable body composition in youth, but the associations seem to be small and somewhat inconsistent^9,11^. While some studies have shown that screen time is more strongly associated with overweight and adiposity than physical activity, others suggest that higher levels of sedentary time are not associated with or predictive of adiposity in youth when taking physical activity into account, or that physical activity attenuates the association between sedentary behaviors and adiposity^9,12,13^.

The association between sedentary behaviors and adiposity may also differ depending on the adiposity-related outcomes used. For example, waist-to-height ratio (WHtR) has been shown to be a better marker of adiposity or predictor of cardiovascular disease risk factors in children than BMI^14–16^. Nevertheless, BMI is the most commonly used outcome in studies examining the relationship between sedentary behaviors and adiposity in children, and a limited number of studies have assessed other adiposity measures^9^. Therefore, the role of the outcome measure remains unclear^9^. In summary, the research evidence on the relationship between sedentary behaviors and other adiposity-related outcomes than BMI in children is still limited, and the role of physical activity in this relationship warrants further investigation.

This study examines the associations of recreational sedentary screen time with overweight and central adiposity among 10,000 children. The specific aims are to examine 1) whether TV viewing and computer use are associated with BMI, 2) whether the associations of TV viewing and computer use with BMI differ according to weekly exercise duration, and 3) whether the associations of TV viewing and computer use are similar with BMI and WHtR.

## Results

### Characteristics

Table 1 shows characteristics of the participants in total and by BMI groups. The BMI groups differed significantly in age, sex, language, sleep duration, WHtR, weekly exercise duration, TV viewing and computer use. The percentage of Heavy TV viewers was higher in the overweight group (46%) than in the normal weight (35%) or underweight groups (31%). Moreover, the percentage of Heavy computer users was higher among the overweight group (43%) than in the normal weight (34%) or underweight groups (29%).

**Table 1:** Characteristics of participants by body mass index groups.

Characteristic	Body mass index groups^a^			Total	*p* value^b^
	Underweight	Normal weight	Overweight		
	n (%) = 1,129 (11.0)	n (%) = 7,556 (73.8)	n (%) = 1,543 (15.2)	n (%) = 10,228 (100.0)	
**Age in years, mean (SD)**	11.2 (0.9)	11.1 (0.8)	11.2 (0.8)	11.1 (0.8)	0.006
**Sex, n (%)**					
Girl	680 (60.2)	3,896 (51.6)	787 (51.0)	5,363 (52.4)	<0.001
Boy	449 (39.8)	3,660 (48.4)	756 (49.0)	4,865 (47.6)	
**Language, n (%)**					
Finnish	1,065 (94.3)	7,033 (93.1)	1,440 (93.3)	9,538 (93.3)	0.029
Swedish	39 (3.5)	338 (4.5)	51 (3.3)	428 (4.2)	
Other	25 (2.2)	185 (2.4)	52 (3.4)	262 (2.6)	
**Sleep duration**^c^**, n (%)**					
Less than recommended	56 (5.0)	545 (7.2)	166 (10.8)	767 (7.5)	<0.001
Recommended	1,051 (93.1)	6,876 (91.0)	1,340 (86.8)	9,267 (90.6)	
More than recommended	22 (1.9)	135 (1.8)	37 (2.4)	194 (1.9)	
**Waist-to-height ratio**^d^**, n (%)**					
Lower tertile	929 (82.3)	2,473 (32.7)	6 (0.4)	3,408 (33.3)	<0.001
Middle tertile	184 (16.3)	3,216 (42.6)	75 (4.9)	3,475 (34.0)	
Upper tertile	16 (1.4)	1,867 (24.7)	1,462 (94.8)	3,345 (32.7)	
**Exercise**^**e**^**, n (%)**					
Low	452 (40.0)	2,563 (33.9)	688 (44.6)	3,703 (36.2)	<0.001
Medium	349 (30.9)	2,336 (30.9)	469 (30.4)	3,154 (30.8)	
High	328 (29.1)	2,657 (35.2)	386 (25.0)	3,371 (33.0)	
**TV viewing**^**f**^**, n (%)**					
Light	429 (38.0)	2,491 (33.0)	348 (22.6)	3,268 (32.0)	<0.001
Medium	348 (30.8)	2,452 (32.5)	484 (31.4)	3,284 (32.1)	
Heavy	352 (31.2)	2,613 (34.6)	711 (46.1)	3,676 (35.9)	
**Computer use**^**g**^**, n (%)**					
Light	417 (36.9)	2,597 (34.4)	398 (25.8)	3,412 (33.4)	<0.001
Medium	381 (33.7)	2,596 (34.4)	481 (31.2)	3,458 (33.8)	
Heavy	331 (29.3)	2,363 (31.3)	664 (43.0)	3,358 (32.8)	

^a^Categorization based on the Cole and Lobstein Classification^45^.

^b^Results from Chi-square test (except for age from ANOVA).

^c^Sleep duration on school nights. Categorized according to the Childhood Sleep Guidelines by the American Academy of Pediatrics^44^.

^d^Waist circumference (cm) divided by height (cm), and categorized into tertiles based on the distribution in the sample.

^e^Categorization based on the responses for a question on exercise duration during leisure-time (low = around five hours a week or less; medium = around six to eight hours a week; high = around nine or ten hours a week).

^f^Categorization based on the distribution of responses for two questions on recreational TV viewing during school days and weekends/days off.

^g^Categorization based on the distribution of responses for two questions on recreational computer use during school days and weekends/days off.

SD, standard deviation.

### The relationship between screen time and body mass index

We compared underweight and overweight with normal weight when we examined the associations between screen time and BMI groups (Table 2). Compared with Light TV viewers, Medium or Heavy TV viewers had a lower risk of being underweight but a higher risk of being overweight. Compared with Light computer users, Heavy computer users had a higher risk of being overweight.

**Table 2:** Odds ratios and confidence intervals for sedentary TV viewing and computer use related to the children’s body mass index categories (n = 10,228).

	Body mass index categories^a^			Risk for Underweight^b^			Risk for Overweight^b^		
	Underweight	Normal weight	Overweight						
	n (%)	n (%)	n (%)	OR	95% CI	*p* value^*c*^	OR	95% CI	*p* value^*c*^
**TV viewing**^**d**^									
Light	429 (38.0)	2,491 (33.0)	348 (22.6)	1			1		
Medium	348 (30.8)	2,452 (32.5)	484 (31.4)	**0.81**	**0.69**–**0.95**	**0.008**	**1.30**	**1.11**–**1.52**	**0.001**
Heavy	352 (31.2)	2,613 (34.6)	711 (46.1)	**0.76**	**0.64**–**0.90**	**0.002**	**1.57**	**1.34**–**1.83**	<**0.001**
**Computer use**^**e**^									
Light	417 (36.9)	2,597 (34.4)	398 (25.8)	1			1		
Medium	381 (33.7)	2,596 (34.4)	481 (31.2)	1.01	0.87–1.19	0.860	1.07	0.92–1.25	0.363
Heavy	331 (29.3)	2,363 (31.3)	664 (43.0)	1.05	0.88–1.26	0.572	**1.42**	**1.21**–**1.67**	<**0.001**

^a^Categorization based on the Cole and Lobstein Classification^45^.

^b^Compared to Normal weight.

^c^Main effects from multinomial logistic regression. Variables in the model: age, sex, language, sleep duration on school nights, weekly exercise, TV viewing and computer use.

^d^Categorization based on the distribution of responses for two questions on recreational TV viewing during school days and weekends/days off.

^e^Categorization based on the distribution of responses for two questions on recreational computer use during school days and weekends/days off.

OR, odds ratio; CI, confidence interval.

We detected interactions between exercise groups and TV viewing (*p* = 0.012) and between exercise groups and computer use (*p* = 0.010). Table 3 shows the associations between screen time and BMI categories stratified by exercise groups Low, Medium and High. Among children in the Low and in the High exercise groups, Medium and Heavy TV viewers and Heavy computer users had a higher risk of being overweight. Among children in the Medium exercise group, only Heavy TV viewers had a higher risk of being overweight. We further examined the associations between screen time and BMI categories (adjusting for same confounders) in children with more extreme exercise levels. The results regarding the risk of being overweight remained similar in children with exercise ≤three hours/week (n = 1,634): Medium (OR: 1.49, 95% CI: 1.04–2.13) and Heavy (OR: 1.71, 95% CI: 1.19–2.44) TV viewers had a higher risk of being overweight; and in children with exercise ≥ten hours/week (n = 2,296): Heavy TV viewers (OR: 1.73, 95% CI: 1.19–2.54) and Heavy computer users (OR: 1.53, 95% CI: 1.04–2.25) had a higher risk of being overweight.

**Table 3:** Odds ratios and confidence intervals for sedentary TV viewing and computer use related to the children’s body mass index groups^a^ stratified by weekly leisure-time exercise duration (n = 10,228).

	Risk for Underweight^b^			Risk for Overweight^b^		
	OR	95% CI	*p* value^*c*^	OR	95% CI	*p* value^*c*^
**Model for children in the Low exercise group (≤5 hours/week), n = 3,703**						
**TV viewing**^**d**^						
Light	1			1		
Medium	0.79	0.61–1.02	0.069	**1.28**	**1.01**–**1.62**	**0.039**
Heavy	**0.70**	**0.54**–**0.92**	**0.010**	**1.46**	**1.15**–**1.85**	**0.002**
**Computer use**^**e**^						
Light	1			1		
Medium	1.15	0.89–1.48	0.286	1.19	0.95–1.50	0.137
Heavy	1.10	0.83–1.47	0.503	**1.37**	**1.08**–**1.76**	**0.010**
**Model for children in the Medium exercise group (6-8 hours/week), n = 3,154**						
**TV viewing**^**d**^						
Light	1			1		
Medium	0.97	0.73–1.29	0.836	1.29	0.97–1.73	0.082
Heavy	0.83	0.60–1.13	0.233	**1.74**	**1.30**–**2.33**	<**0.001**
**Computer use**^**e**^						
Light	1			1		
Medium	0.88	0.66–1.17	0.388	1.00	0.76–1.32	0.994
Heavy	1.00	0.72–1.38	0.988	1.33	0.99–1.78	0.056
**Model for children in the High exercise group (9-10 hours/week), n = 3,371**						
**TV viewing**^**d**^						
Light	1			1		
Medium	**0.69**	**0.51**–**0.92**	**0.012**	**1.37**	**1.02**–**1.83**	**0.037**
Heavy	0.80	0.58–1.10	0.168	**1.55**	**1.14**–**2.12**	**0.005**
**Computer use**^**e**^						
Light	1			1		
Medium	1.02	0.76–1.35	0.919	1.00	0.75– 1.34	0.998
Heavy	1.02	0.72–1.44	0.927	**1.62**	**1.19**–**2.21**	**0.002**

Likelihood ratio test to evaluate models with and without interaction between exercise and TV viewing: *p* = 0.012.

Likelihood ratio test to evaluate models with and without interaction between exercise and computer use: *p* = 0.010.

^a^Categorization based on the Cole and Lobstein Classification^45^.

^b^Compared to Normal Weight.

^c^Main effects from multinomial logistic regression. Variables in the model: age, sex, language, sleep duration on school nights, TV viewing and computer use.

^d^Categorization based on the distribution of responses for two questions on recreational TV viewing during school days and weekends/days off.

^e^Categorization based on the distribution of responses for two questions on recreational computer use during school days and weekends/days off.

OR, odds ratio; CI, confidence interval.

### The relationship between screen time and waist-to-height ratio

We repeated all analyses using WHtR tertiles instead of BMI categories. The WHtR tertiles differed significantly in age, sex, sleep duration, BMI, exercise, TV viewing and computer use, but not in language. The percentages of Heavy TV viewers were 43%, 34% and 31% in the Upper, Middle and Lower WHtR tertiles, respectively. The percentages of Heavy computer users, in turn, were 40%, 31% and 29%, respectively (see Supplementary Table S1).

When examining the associations between screen time and WHtR tertiles, we used the Middle tertile as a reference group. Compared with Light TV viewers, Heavy TV viewers had a lower risk of being in the Lower WHtR tertile (adjusted OR: 0.86, 95% CI: 0.76–0.99). Furthermore, Medium and Heavy TV viewers had a higher risk of being in the Upper WHtR tertile (adjusted OR: 1.27, 95% CI: 1.12–1.44 and adjusted OR: 1.48, 95% CI: 1.29–1.69, respectively). Compared with Light computer users, only Heavy computer users had a higher risk of being in the Upper WHtR tertile (adjusted OR: 1.29, 95% CI: 1.13–1.49) (see Supplementary Table S2).

Moreover, we observed significant interactions between exercise groups and TV viewing (*p* = 0.002) and between exercise groups and computer use (*p* = 0.001). Among children in the Low exercise group, Medium and Heavy TV viewers and Heavy computer users had a higher risk of being in the Upper WHtR tertile. Among children in the Medium exercise group, Medium and Heavy TV viewers had a higher risk of being in the Upper WHtR tertile. Finally, among children in the High exercise group, Heavy TV viewers and Heavy computer users had a higher risk of being in the Upper WHtR tertile (see Supplementary Table S3).

## Discussion

This cross-sectional study examined the associations of recreational TV viewing and computer use, performed sitting down, with overweight and central adiposity in a large sample of Finnish children. We showed that greater amounts of TV viewing and computer use were associated with being overweight after adjusting for several confounding factors. Moreover, Heavy TV viewers had a higher risk of being overweight regardless of leisure-time exercise duration. Finally, TV viewing and computer use showed similar associations with overweight assessed by BMI and central adiposity assessed by WHtR. To summarize, heavy TV viewers (broadcast, online etc.) during free time are the heaviest regardless of how much they exercise during free time.

Our results regarding TV viewing are in accordance with previous mostly cross-sectional and few longitudinal studies reporting associations between greater amounts of TV viewing and overweight or adiposity among children^17,20^. The amount of TV viewing showed stronger associations with BMI categories than the amount of computer use in our study. This observation is in line with systematic reviews reporting positive associations between TV viewing and overweight or adiposity, but a less clear association for computer use^9,21^.

Possible reasons for the stronger association for TV viewing include that TV viewing may have a lower energy expenditure compared with computer use. Exergames (i.e., electronic games involving physical exertion) can increase energy expenditure over the resting state or be comparable to physical activity of moderate intensity^22,23^. In addition, the psychological stimulus or excitement alone may increase energy expenditure during exergaming^24^. In our study, however, the children reported computer use performed sitting down. Nevertheless, energy expenditure has been reported to be higher during seated gaming compared with watching video on a TV in a supine position^25^. Moreover, children have been shown to not only have somewhat higher wrist-based but also thigh-based accelerometer counts, as well as higher heart rates when playing sedentary computer games compared with watching movies^26^. Other possible explanations for the stronger relationship between TV viewing and being overweight include that the children are more exposed to unhealthy food advertising when watching TV than when using the computer, which may stimulate more frequent and unhealthy food intake^19,27^. In addition, TV viewing may be easier to recall than computer use, which may account for stronger or more consistent associations found in the previous studies as well as in our study.

We observed that heavy TV viewing was associated with being overweight regardless of the weekly leisure-time exercise duration of the children, suggesting that among those with high screen times, exercise habits may not override the negative effects of sedentary behaviors. Previous studies have shown conflicting results concerning the modifying effect of physical activity or exercise in the relationship between sedentary behaviors and adiposity in children^9,12,13^. In accordance with our findings, some studies have demonstrated associations of sedentary behaviors with adiposity independently of physical activity, while others have not^9,12^. In our study, greater amounts of screen time were associated with overweight and central adiposity across different exercise groups, even among children with the highest exercise level.

Greater amounts of TV viewing were associated with a higher risk of being overweight but also with a lower risk of being underweight in our study, indicating a linear relationship between TV viewing and BMI. Only a few previous studies have examined the relationship between screen time and underweight. In contrast to our results, a study of more than 25,000 Canadian adolescents reported that greater screen time was associated with a higher risk of being underweight in boys^20^. Similarly, another study with 53,769 Korean adolescents showed that more leisure-time sitting (watching TV, playing videogames, internet etc.) was associated with a higher risk of being underweight^28^. One of these studies considered children with a ≤5 percentile BMI as underweight and adjusted their analyses for age and sex^20^, whereas the other study considered <5 percentile BMI as underweight and adjusted for several confounding factors including socioeconomic status and physical activity, but as a dichotomous variable (low/high)^28^. We used the IOTF age- and sex-specific BMI categories and WHtR tertiles and slightly different confounders, which may explain the dissimilar results between the previous studies and our study.

BMI is the most widely used outcome measure in studies examining the relationship between sedentary behavior and being overweight among youth^9^. We repeated our analyses with WHtR, a measure of central adiposity, and the findings were similar to those of BMI; Medium and Heavy TV viewers and Heavy computer users had a higher risk of being in the Upper WHtR tertile, and Heavy TV viewers had a higher risk of being in the Upper WHtR tertile regardless of the exercise duration. In accordance with a few previous studies, our findings indicate that the association between screen time and adiposity is not dependent on the weight-related measure used^9^. In contrast, some studies have not observed associations of sedentary behavior with body fatness and waist circumference^9,29^. A cross-sectional study on 507 Canadian children aged 9 to 11 years did not find an association between accelerometer-assessed sedentary time or self-reported screen time and WHtR after adjusting for accelerometer-assessed moderate-to-vigorous-intensity physical activity^30^.

Limitations of our study include that we did not assess sedentary time and physical activity with a device, such as an accelerometer, but with a questionnaire. However, the exercise questions we used have been validated against an accelerometer, with results showing that they are suitable for classifying children according to their exercise level^31^. Moreover, similar screen time questions that we used have shown acceptable reliability in school-aged children^32–34^. Regarding validation of the questions, there is a lack of a ‘gold standard’ reference measure for self-reported sedentary behaviors^35,36^. Additional limitations of our screen time measures include that TV viewing and computer use can occur simultaneously, and that bout duration cannot be accounted for^35^. Self-report tools, however, enable to assess the type of sedentary behavior (for example screen time) and are more feasible in large-scale studies. Our data were collected between the years 2011 and 2014. Since that time technology and the use of screen-based devices have changed, for example watching TV shows on a computer, tablet or smartphone is partly replacing traditional TV viewing. Nevertheless, the phenomenon of sedentary screen time remains existing.

A further limitation is that we only assessed the children’s exercise behavior during leisure-time and not their total physical activity. However, we similarly assessed the children’s screen time behavior during leisure-time, and thus were able to examine the associations of children’s free time behavior with adiposity. We only assessed exercise duration without intensity, and were not able to take into account the variations in physical activity or screen time on the way to or at school, or during breaks at school. Screen time during the school day is a potential confounding factor, because it can affect screen time during the following leisure-time^37^. Anyhow, physical activity education is included in the Finnish national core curriculum for basic education, and thus the amount and quality of grade-specific physical education lessons should be the same in all Finnish schools. We did not adjust the analyses for all possible confounding factors, such as dietary intakes. A systematic review showed, however, that the associations between sedentary behavior, such as TV viewing, and adiposity among youth are largely independent of diet^21^. Another limitation is the cross-sectional study design, thus, we cannot draw any conclusions on causality. It may be that heavy sedentary screen use leads to becoming overweight, or that being overweight increases the likelihood of heavy screen use. The relationship between screen time and overweight may also be reciprocal. In our study, 2.6% of the children were obese but 15.2% were either overweight or obese. It is important to examine the factors associated with overweight and not only obesity, hence overweight children are more likely to stay overweight or become obese, and suffer from adverse health consequences in the future^38,39^.

The strengths of our study include that we examined a large sample of children. Moreover, we assessed recreational screen time by combining information on TV viewing and computer use both during school days and during weekends. Many of the previous studies have reported screen time only during school days^9^. With our computer question we assessed time playing sedentary computer games but we did not assess time playing active video games. So, we assessed sedentary screen time, which can be seen as a proxy for sitting. A further strength is that we adjusted the analyses for several possible confounding factors, including exercise and sleep duration, which have been suggested to be among the key potential confounders in the relationship between sedentary behaviors and adiposity in children^9^. In the Fin-HIT cohort, we were able to address the risk of being underweight as well. This further supported an association between screen time and body size, suggesting a linear relationship. Another strength of our study is that we measured the children’s weight, height and waist circumference and examined the associations of screen time with both BMI and WHtR to strengthen the reliability of the results. Few previous studies have adopted standardized methods to assess actual body fatness or waist circumference^9^. Finally, Our results can be generalized to Finnish children^40^, and at least to some extent to children in other Western countries.

## Conclusions

Children with high amounts of recreational sedentary TV viewing and computer use have a higher risk for overweight and central adiposity. Furthermore, High amounts of TV viewing seem to be detrimental regardless of leisure-time exercise behavior. Considering that screen time is highly prevalent in modern society, and not least among children, strategies to reduce sedentary screen time could be effective in curbing the alarmingly high rates of overweight and obesity among youth.

## Methods

### Study design

This analysis is part of the Finnish Health in Teens study (Fin-HIT), a cohort including children aged around 9 to 12 years at enrollment^40^. Altogether 38,000 children were invited to participate from 496 schools and 44 municipalities in Finland between the years 2011 and 2014. Depending on the school, all children from the Finnish grades 3, 4, 5 and/or 6 were invited with no exclusion criteria. A total of 11,407 children (30% of the invited) participated. The study design, recruitment methods and the cohort have been described in more detail elsewhere^40^. In this analysis, we include 10,228 participants with information available on TV viewing, computer use, BMI, WHtR and the relevant covariates. The Coordinating Ethics Committee of the Hospital District of Helsinki and Uusimaa in Finland has approved the study protocol (169/13/03/00/10), and the children and their guardians have signed an informed consent form. All study procedures adhered to the 1964 Helsinki Declaration and its later amendments, or comparable ethical standards.

### Measurements

#### Recreational sedentary screen time

**Figure 1:**
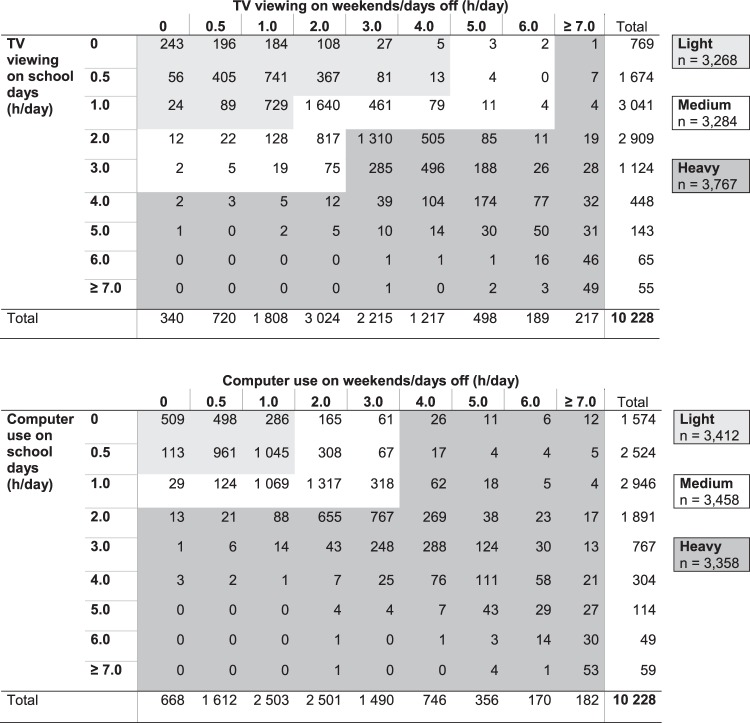
Cross tabulations illustrate the recategorization of participants according to time spent on sedentary TV viewing and computer use into three approximately similar sized groups: Light, Medium and Heavy TV viewers/computer users.

The children used an electronic tablet at school to answer a web-based questionnaire including health- and lifestyle-related questions. We assessed screen time with questions adapted from the World Health Organisation Health Behaviour in School-aged Children (WHO HBSC) study^41^. The screen time questions in the WHO HBSC study have shown fair to substantial test–retest reliability depending on the evaluation criteria used^32–34^. The content validity of self-reported screen time measures, in turn, is considered difficult to assess against more objective measures because for example accelerometers measure sedentary time but not the type of sedentary behavior^36^. We assessed TV viewing with the question: ‘How many hours a day during your free time do you normally watch TV, videos or DVDs? By TV, we mean programs that can be watched on TV as well as on a computer.’ Further, we assessed computer use with the question: ‘How many hours a day during your free time do you normally use a computer, e.g. spend time on the Internet, chat or play computer or TV games sitting down (e.g., PlayStation, Xbox)?’ We asked the questions about TV viewing and computer use separately for ‘school days’ and for ‘weekends or days off’. Both questions had nine response options ranging from ‘I do not watch TV, videos or DVDs/I do not use a computer’ to ‘Around seven hours a day or more’. For the analyses, TV viewing and computer use hours/day for ‘school days’ and ‘weekends or days off’ were combined by using a cross tabulation of the variables. We recategorized the children into three groups (Light, Medium and Heavy TV viewers/computer users) with approximately similar numbers of participants (Fig. 1). We were unable to divide the children into exact tertiles because the children answered the questions by choosing from nine response options, as illustrated in Fig. 1.

#### Exercise

We assessed leisure-time exercise with a question adapted from the Finnish School Study^42^: ‘How many hours a week do you normally exercise or do sports during your free time? Include all the exercise you do in a club or team and any exercise by yourself, with family or friends. Do not count any exercise at school or on the way to school.’ The question included ten response options ranging from ‘An hour a week or less’ to ‘Around ten hours a week’. We recategorized the responses based on the sample distribution to obtain three groups with approximately similar numbers of participants. The final three exercise duration groups are 1) Low (up to around five hours a week), 2) Medium (around six to eight hours a week), and 3) High (around nine or ten hours a week). In addition, we repeated a part of the analyses in children with more extreme exercise levels: ≤three hours/week; and ≥ten hours/week. A previous validation study showed that the children in the Fin-HIT study who reported higher exercise had higher accelerometer-measured physical activity; the exercise questions used had a moderate capability to categorize children according to their physical activity levels^31^.

#### Sleep duration

We asked the participants about falling asleep on school nights with the question: ‘When do you usually fall asleep in the evenings on a school night?’ with 12 response options. Further, we asked about waking up on school days with a question: ‘When do you usually wake up on school days?’ with 7 response options. The questions were adapted from the questions used in the LifeGene, WHO HBSC and Finnish School Study^41–43^. We calculated sleeping duration on school nights based on the responses and categorized the durations into three groups: 1) less than recommended, 2) recommended and 3) more than recommended. Sleep duration was categorized according to the age-specific Childhood Sleep Guidelines by the American Academy of Pediatrics^44^. Children 6 to 12 years of age should sleep 9–12 hours per 24 hours, and teenagers 13 to 18 years of age 8–10 hours per 24 hours on a regular basis to promote optimal health^44^.

#### Anthropometrics

Trained field workers visited the schools and measured the children’s height (cm), weight (kg) and waist circumference (cm), as described elsewhere^40^. We calculated BMI and categorized the children as underweight, normal weight, overweight or obese according to age- and sex-specific cut-offs from the International Obesity Task Force (IOTF) classification^45^. For the analyses, we combined the BMI groups of overweight and obese (group “overweight”) because the number of obese children was too small (2.6%). We divided the measured waist circumference by the measured height to calculate WHtR as a marker of central adiposity and a predictor of cardiovascular disease risk factors in children^14–16^. For the analyses, we categorized WHtR into tertiles: 1) Lower, 2) Middle and 3) Upper.

#### Demographics

Parents reported the children’s age, gender and language spoken at home, and we confirmed those by linking our data with the National Population Information System at the Population Register Center.

### Statistical analyses

The differences in participant characteristics between BMI categories and WHtR tertiles were examined with Chi-square test or ANOVA (adjusted by Brown–Forsythe when appropriate). Missing data for language (n = 3), sleep (n = 39) and exercise (n = 127) was identified, and participants with missing data were excluded from further analyses. Associations between BMI (and WHtR) and screen time were examined using multinomial logistic regression in order to calculate odds ratios (OR) with 95% confidence intervals (CI). BMI categories (and WHtR tertiles) were treated as dependent variables, and screen time variables as independent variables in the analyses. In all analyses, extreme categories of BMI (or WHtR tertiles) were compared with normal weight (or the Middle WHtR tertile). The analyses were adjusted for sex (girl/boy), language spoken at home (Finnish/Swedish/other), sleep duration on school nights (less than recommended, recommended, more than recommended) and leisure-time exercise group (Low, Medium, High). Physical activity/exercise and sleep have been suggested to be among the key potential confounders in the relationship between sedentary behaviors and adiposity in children^9^. The interaction between exercise and BMI or WtHR was assessed using the likelihood ratio test, comparing models with and without the respective interaction terms. We performed the analyses for all children, and stratified by exercise groups. A 5% significance level was chosen. The analyses were conducted with the IBM SPSS Statistics software version 25 (Armonk, NY: IBM Corp.) and SAS 9.4 software (SAS Institute, Inc., Cary, North Carolina).

### Disclaimer

Where authors are identified as personnel of the International Agency for Research on Cancer/World Health Organization, the authors alone are responsible for the views expressed in this article and they do not necessarily represent the decisions, policy or views of the International Agency for Research on Cancer/World Health Organization.

## Supplementary information

Supplementary Tables S1-S3

## Data Availability

The datasets generated during and/or analysed during the current study are available from the corresponding author on reasonable request.
