# Supplementary material for: Heavy screen users are the heaviest among 10,000 children
Source: Sci Rep. 2019 Aug 1;9:11158. doi: 10.1038/s41598-019-46971-6 (PMC6673695; doi:10.1038/s41598-019-46971-6)
Supplement: Supplementary file 1 — Supplementary Tables S1-S3 [file 41598_2019_46971_MOESM1_ESM.pdf]

Heavy screen users are the heaviest among 10,000 children

Engberg E, Figueiredo RAO, Rounge TB, Weiderpass E, Viljakainen H

Supplementary Table S1 Characteristics of participants by waist-to-height ratio tertiles.

Characteristic	Waist-to-height ratio[a]			Total	*p* value[b]
	**Lower tertile** 0.2685 – 0.4116	**Middle tertile** 0.4117 – 0.4429	**Upper tertile** 0.4430 – 0.7733		
	n (%) = 3,408 (33.3)	n (%) = 3,475 (34.0)	n (%) = 3,345 (32.7)	n (%) = 10,228 (100.0)	
**Age in years, mean (SD)**	11.2 (0.8)	11.1 (0.9)	11.1 (0.8)	11.1 (0.8)	< 0.001
**Sex, n (%)**					
Girl	2,229 (65.4)	1,650 (47.5)	1,484 (44.4)	5,363 (52.4)	< 0.001
Boy	1,179 (34.6)	1,825 (52.5)	1,861 (55.6)	4,865 (47.6)	
**Language, n (%)**					
Finnish	3,174 (93.1)	3,261 (93.8)	3,103 (92.8)	9,538 (93.3)	0.179
Swedish	148 (4.3)	140 (4.0)	140 (4.2)	428 (4.2)	
Other	86 (2.5)	74 (2.1)	102 (3.0)	262 (2.6)	
**Sleep duration[c], n (%)**					
Less than recommended	229 (6.7)	242 (7.0)	296 (8.8)	767 (7.5)	0.002
Recommended	3,122 (91.6)	3,170 (91.2)	2,975 (88.9)	9,267 (90.6)	
More than recommended	57 (1.7)	63 (1.8)	74 (2.2)	194 (1.9)	
**Body mass index groups[d], n (%)**					
Underweight	929 (27.3)	184 (5.3)	16 (0.5)	1,129 (11.0)	< 0.001
Normal weight	2,473 (72.6)	3,216 (92.5)	1,867 (55.8)	7,556 (73.9)	
Overweight + obese	6 (0.2)	76 (2.2)	1,462 (43.7)	1,543 (15.1)	
**Exercise[e], n (%)**					
Low	1,172 (34.4)	1,169 (33.6)	1,362 (40.7)	3,703 (36.2)	< 0.001
Medium	1,068 (31.3)	1,057 (30.4)	1,029 (30.8)	3,154 (30.8)	
High	1,168 (34.3)	1,249 (35.9)	954 (28.5)	3,371 (33.0)	
**TV viewing[f], n (%)**					
Light	1,247 (36.6)	1,184 (34.1)	837 (25.0)	3,268 (32.0)	< 0.001
Medium	1,101 (32.3)	1,115 (32.1)	1,068 (31.9)	3,284 (32.1)	
Heavy	1,060 (31.1)	1,176 (33.8)	1,440 (43.0)	3,676 (35.9)	
**Computer use[g], n (%)**					
Light	1,320 (38.7)	1,190 (34.2)	902 (27.0)	3,412 (33.4)	< 0.001
Medium	1,120 (32.9)	1,224 (35.2)	1,114 (33.3)	3,458 (33.8)	
Heavy	968 (28.4)	1,061 (30.5)	1,329 (39.7)	3,358 (32.8)	

[a] Waist circumference (cm) divided by height (cm), and categorized into tertiles based on the distribution in the sample

[b] Results from Chi-square test (except for age from ANOVA adjusted by Brown–Forsythe)

[c] Sleep duration on school nights. Categorized according to the Childhood Sleep Guidelines by the American Academy of Pediatrics[44]

[d] Categorization based on the Cole and Lobstein Classification[45]

[e] Categorization based on the responses for a question on exercise duration during leisure-time (Low = around five hours a week or less; Medium = around six to eight hours a week; High = around nine or ten hours a week)

[f] Categorization based on the distribution of responses for two questions on leisure-timel TV viewing during school days and weekends / days off

[g] Categorization based on the distribution of responses for two questions on leisure-time computer use during school days and weekends / days off

SD, standard deviation

Supplementary Table S2 Odds ratios and confidence intervals for TV viewing and computer use related to the children's waist-to-height ratio tertiles (n = 10,228).

	**Waist-to-height ratio tertiles[a]**			**Risk for Lower tertile[b]**			**Risk for Upper tertile[b]**		
	**Lower tertile**	**Middle tertile**	**Upper tertile**						
	**n (%)**	**n (%)**	**n (%)**	**OR**	**95% CI**	***p* value[c]**	**OR**	**95% CI**	***p* value[c]**
**TV viewing[d]**									
Light	1,247 (36.6)	1,184 (34.1)	837 (25.0)	1			1		
Medium	1,101 (32.3)	1,115 (32.1)	1,068 (31.9)	0.96	0.85 – 1.08	0.472	**1.27**	**1.12 – 1.44**	**< 0.001**
Heavy	1,060 (31.1)	1,176 (33.8)	1,440 (43.0)	**0.86**	**0.76 – 0.99**	**0.030**	**1.48**	**1.29 – 1.69**	**< 0.001**
**Computer use[f]**									
Light	1,320 (38.7)	1,190 (34.2)	902 (27.0)	1			1		
Medium	1,120 (32.9)	1,224 (35.2)	1,114 (33.3)	0.94	0.83 – 1.06	0.297	1.06	0.94 – 1.21	0.334
Heavy	968 (28.4)	1,061 (30.5)	1,329 (39.7)	1.05	0.91 – 1.20	0.531	**1.29**	**1.13 – 1.49**	**< 0.001**

[a] Waist circumference (cm) divided by height (cm), and categorized into tertiles based on the distribution in the sample

[b] Compared to Middle tertile

[c] Main effects from multinomial logistic regression. Variables in the model: age, sex, language, sleep duration on school nights, physical activity, TV viewing and computer use

[d] Categorization based on the distribution of responses for two questions on leisure-time TV viewing during school days and weekends / days off

[e] Categorization based on the distribution of responses for two questions on leisure-time computer use during school days and weekends / days off

OR, odds ratio; CI, confidence interval

Supplementary Table S3 Odds ratios and confidence intervals for TV viewing and computer use related to the children's waist-to-height ratio tertiles[a] stratified by weekly leisure-time exercise duration (n = 10,228).

	Risk for Lower tertile[b]			Risk for Upper tertile[b]		
	**OR**	**95% CI**	***p* value[c]**	**OR**	**95% CI**	***p* value[c]**
**Model for children in the Low exercise group (≤ 5 hours/week), n = 3,703**						
**TV viewing[d]**						
Light	1			1		
Medium	0.94	0.76 – 1.16	0.534	**1.35**	**1.10 – 1.67**	**0.005**
Heavy	0.82	0.66 – 1.02	0.071	**1.32**	**1.07 – 1.64**	**0.011**
**Computer use[e]**						
Light	1			1		
Medium	1.09	0.88 – 1.34	0.435	1.12	0.91 – 1.38	0.277
Heavy	**1.28**	**1.01 – 1.62**	**0.040**	**1.50**	**1.19 – 1.87**	**< 0.001**
**Model for children in the Medium exercise group (6-8 hours/week), n = 3,154**						
**TV viewing[d]**						
Light	1			1		
Medium	1.09	0.87 – 1.35	0.450	**1.33**	**1.05 – 1.68**	**0.018**
Heavy	0.97	0.76 – 1.23	0.804	**1.90**	**1.49 – 2.42**	**< 0.001**
**Computer use[e]**						
Light	1			1		
Medium	0.88	0.71 – 1.10	0.266	1.07	0.85 – 1.35	0.565
Heavy	0.95	0.74 – 1.22	0.676	1.02	0.79 – 1.31	0.897
**Model for children in the High exercise group (9-10 hours/week), n = 3,371**						
**TV viewing[d]**						
Light	1			1		
Medium	0.89	0.73 – 1.10	0.283	1.17	0.94 – 1.46	0.165
Heavy	0.85	0.68 – 1.07	0.164	**1.33**	**1.04 – 1.69**	**0.021**
**Computer use[e]**						
Light	1			1		
Medium	0.87	0.71 – 1.06	0.168	1.00	0.80 – 1.24	0.988
Heavy	0.91	0.71 – 1.16	0.432	**1.41**	**1.10 – 1.80**	**0.007**

Likelihood ratio test to evaluate models with and without interaction between physical activity and TV viewing: $p = 0.002$

Likelihood ratio test to evaluate models with and without interaction between physical activity and computer use: $p = 0.001$

[a] Waist circumference (cm) divided by height (cm), and categorized into tertiles based on the distribution in the sample

[b] Compared to Middle tertile

[c] Main effects from multinomial logistic regression. Variables in the model: age, sex, language, sleep duration on school nights, TV viewing and computer use

[d] Categorization based on the distribution of responses for two questions on leisure-time TV viewing during school days and weekends / days off

[e] Categorization based on the distribution of responses for two questions on leisure-time computer use during school days and weekends / days off

OR, odds ratio; CI, confidence interval
